# Effect of coercive measures on mental health status in adult psychiatric populations: a nationwide trial emulation

**DOI:** 10.1017/S2045796024000416

**Published:** 2024-09-12

**Authors:** S. Baggio, S. Kaiser, C.G. Huber, A. Wullschleger

**Affiliations:** 1 Institute of Primary Health Care (BIHAM), University of Bern, Bern, Switzerland; 2 Laboratory of Population Health (#PopHealthLab), University of Fribourg, Fribourg, Switzerland; 3 Adult Psychiatry Division, Department of Psychiatry, Geneva University Hospitals, Geneva, Switzerland; 4 University Psychiatric Clinics (UPK), University of Basel, Basel, Switzerland

**Keywords:** causal inference, coercive measures, psychiatric hospitalizations

## Abstract

**Aims:**

Healthcare staff use coercive measures to manage patients at acute risk of harm to self or others, but their effect on patients’ mental health is underexplored. This nationwide Swiss study emulated a trial to investigate the effects of coercive measures on the mental health of psychiatric inpatients at discharge.

**Methods:**

We analysed retrospective longitudinal data from all Swiss adult psychiatric hospitals that provided acute care (2019–2021). The primary exposure was any coercive measure during hospitalization; secondary exposures were seclusion, restraint and forced medication. Our primary outcome was Health of the Nations Outcome Scale (HoNOS) score at discharge. We used inverse probability of treatment weighting to emulate random assignment to the exposure.

**Results:**

Of 178,369 hospitalizations, 9.2% (*n* = 18,800) included at least one coercive measure. In patients exposed to coercive measures, mental health worsened a small but statistically significant amount more than in non-exposed patients. Those who experienced at least one coercive measure during hospitalization had a significantly higher HoNOS score (1.91-point, *p* < .001, 95% confidence interval [CI]: 1.73; 2.09) than those who did not experience any coercive measure. Results were similar for seclusion (1.60-point higher score, *p* < .001, 95% CI: 1.40; 1.79) and forced medication (1.97-point higher score, *p* < .001, 95% CI: 1.65; 2.30). Restraint had the strongest effect (2.83-point higher score, *p* < .001, 95% CI: 2.38; 3.28).

**Conclusions:**

Our study presents robust empirical evidence highlighting the detrimental impact of coercive measures on the mental health of psychiatric inpatients. It underscores the importance of avoiding these measures in psychiatric hospitals and emphasized the urgent need for implementing alternatives in clinical practice.

## Introduction

When patient under treatment for mental health condition acutely endanger themselves or others, staff members at treatment facilities may implement coercive measures that restrict a patient’s freedom of movement or impose a psychopharmacological treatment even when a patient resists. Coercive measures include seclusion (confinement in a closed room), mechanical restraint (e.g., binding a patient to bed rails, restraining them with belts or immobilizing them with physical force) and forced medication. Healthcare staff may use coercive measures to manage a patient’s extreme agitation, aggression, violence, self-harm and life-threatening behaviours (Newton-Howes, 2013), usually in inpatient psychiatric settings, though use varies widely across countries and institutions (Välimäki *et al.*, 2019). Coercive measures encompass interventions from forced medication to interventions with no primary therapeutic purpose except control and restrain (Steinert, 2016). Few nationwide studies estimate the prevalence of coercive measures, but estimates from previous studies suggest that coercive measures are widespread (Lepping *et al.*, 2016; Välimäki *et al.*, 2019).

Despite widespread use, coercive measures pose legal and ethical dilemmas because they violate fundamental human rights and ethical principles of healthcare: autonomy; freedom of movement and will; and bodily integrity (Chieze *et al.*, 2021). There is also growing concern that use of coercive measures during hospitalization harms patients (Chieze *et al.*, 2019). Coercive measures, especially mechanical restraint, have been associated with physical harm or even death (Kersting *et al.*, 2019). A recent systematic review reported that chemical restraint effectively and quickly calmed or sedated the patient, but caused adverse events (e.g., cardiac problems, blood pressure change, oxygen desaturation, intense sedative effect) (Muir-Cochrane *et al.*, 2020). The review suggested using chemical restraint as a last resort. But few studies have rigorously investigated the effects of coercive measures on quantifiable clinical outcomes, especially those related to mental health. Randomized controlled trials (RCTs) and controlled trials suggested no therapeutic benefits of the use of coercive measures (Bergk *et al.*, 2011; Steinert *et al.*, 2013), or even detrimental outcomes, such as high levels of post-traumatic symptoms (Whitecross *et al.*, 2013; Wullschleger *et al.*, 2021). Observational studies also reported no (Kjellin and Wallsten, 2010; Seo *et al.*, 2013) or negative (Baggio *et al.*, 2023) associations between the use of coercive measures and mental health variables. However, these observational studies suffered from serious methodological limitations for determining causality, including cross-sectional design, selection bias, lack of power and lack of confounding adjustment.

We set out to address these research gaps and increase evidence on the mental health effects of coercive measures by conducting a trial emulation based on observational data from the whole Switzerland. Trial emulation mimics an RCT and allows causal inference (Hernán and Robins, 2016), so we used it to test the effect of coercive measures during hospitalization for any reason and on the effects of separate types of coercive measures (seclusion, restraint and forced medication) on mental health status, as assessed by the Health of the Nations Outcome Scale (HoNOS) at hospital discharge. Given the smaller sample sizes for each type of coercive measures, these analyses should be considered exploratory.

## Materials and methods

### Study design and setting

As a first step in trial emulation, we designed an ideal trial: a prospective, open-label, two-arm, parallel group, superiority RCT to compare use and non-use of coercive measures on mental health status at hospital discharge (see Table 1 for this target trial and the corresponding emulated trial). Our emulation was based on retrospective observational data collected by the Swiss National Association for Quality Development in Hospitals and Clinics (ANQ), which monitors quality indicators of hospital care (including somatic, psychiatric and rehabilitation services) throughout Switzerland. The ANQ monitors the use of coercive measures, a key quality indicator. We collected data from all Swiss hospitals and clinics for adult psychiatry that provided acute psychiatric care between 1 January 2019 and 31 December 2021, if they had taken coercive measures (43 hospitals). We retrieved longitudinal data, including measures at hospital admission and discharge.

**Table 1.** Description of the target and emulated trial

Component	Target trial	Emulated trial
Aim	Estimate the relative effect of coercive measures on mental health status in patients hospitalized in adult psychiatry.	Same.
Design	Prospective open-label two-arm parallel superiority randomized trial.	Retrospective cohort study.
Eligibility criteria	*Inclusion criteria:*	*Inclusion criteria:*
	Age ≥18.	Same.
	Being hospitalized in a Swiss hospital between January 2019 and December 2021.	
	*Exclusion criteria:*	*Exclusion criteria:*
	Did not consent to participate.	Refusal to reuse of data for research purposes, missing values on exposure variable.
Treatment strategies	(1) Use of coercive measures during hospitalization	Patients are assigned to the group (1) or (2) if they received or not coercive measures during hospitalization.
	(2) No use of coercive measures during hospitalization.	
Assignment procedures	Participants randomly assigned to either strategy at hospital admission and aware of the strategy they are assigned to.	Randomization is emulated via adjustment for all hypothesized confounding factors identified a priori.
Follow-up	*Start*: Time of treatment assignment (hospital admission).	*Start*: Hospital admission.
	*Stop*: Hospital discharge.	*Stop*: Same.
Outcomes	*Primary outcome*: HoNOS at discharge	Same.
Causal contrasts	ITT effect.	Observational analogue of PP effect.
	PP effect.	
Analysis plan	*ITT*: Compare means between randomized groups.	*PP*: Same as PP analysis.
	*PP*: Compare means between groups receiving/not receiving the treatment, with patients who deviate from protocol being censored and use of inverse probability weighting to adjust for baseline covariates and attrition.	

HoNOS: Health of the Nations Outcome Scale, ITT: intention to treat, PP: per protocol.

The authors assert that all procedures contributing to this work comply with the ethical standards of the relevant national and institutional committees on human experimentation and with the Helsinki Declaration of 1975, as revised in 2013. All procedures involving human subjects/patients were approved by the ethics committee of the canton of Geneva, Switzerland (no. 2021-00263). Since data were anonymized, the study did not require patient consent.

### Participants and in/exclusion criteria

Participants were eligible for study participation if they were at least 18 years old and had been admitted to an adult or geriatric acute psychiatric unit. Participants were excluded if they had not been diagnosed with a psychiatric disorder (Sections F and G3 of International Statistical Classification of Diseases and Related Health Problems 10th Revision, ICD-10) (World Health Organization, 2004) or if they had been admitted to a forensic psychiatric unit.

### Exposure

Participants were classed by whether they received a coercive measure during hospitalization (seclusion, mechanical restraint, forced medication). The legislation requires that coercive measures are only applied to involuntarily admitted patients. However, the initial voluntary admission status can be modified during hospitalization, when such measures are needed. Hence, all patients could receive coercive measures, regardless of their initial admission status. Because data on number of episodes and duration may not have been reliably collected, we did not consider them. We derived a primary exposure and three secondary exposures.

*Primary exposure*: We calculated a binary composite exposure, based on whether any type of coercive measures (seclusion, restraint, forced medication) had been used during hospitalization (yes/no).

*Secondary exposures*: We calculated separate binary exposures for (1) being secluded (confinement in a closed room), (2) being restrained (e.g., using bed rails, belts, tablets or immobilizing the patient by physical force) and (3) receiving forced medication (yes/no).

Swiss federal law limits use of coercive measures to patients who exhibit aggressive behaviour towards others, severely disrupt ward community life or pose a serious risk of harm to self or others. Acute suicidal risk is a considered to be a contraindication for use of seclusion in many hospitals. These measures must be duly prescribed by a psychiatrist, often a senior physician. The duration of prescription and required monitoring differ between cantons and institutions. Forced medication can be prescribed in emergency situations in case of serious endangerment of oneself or others, or on a regular basis to treat the underlying psychiatric condition. Patients can appeal to the civil court against all coercive measures within 10 days.

### Outcome

Our primary outcome was total HoNOS score at discharge. The HoNOS, which comprises 12 items rated on a 0–4 scale, assesses the severity of psychiatric illnesses and their effects on social functioning. The total score can range from 0 to 48; higher scores indicate worse mental health issues (Wing *et al.*, 1998). HoNOS is routinely used for all patients in all psychiatric hospitals and clinics in Switzerland at admission and discharge.

### Confounding factors

Since measuring confounders is critical in an emulated trial, we used existing evidence to identify these relevant confounders (Baggio *et al.*, 2023): age; sex; nationality (Swiss versus other); marital status (coded in three categories: single, divorced or widowed; married or in a registered partnership; and unknown); psychiatric hospitalization during the previous 12 months (yes/no); compulsory admission (yes/no); psychiatric unit (adult versus geriatric); length of hospitalization (under or over 3 weeks); type of primary psychiatric disorder (as done in a previous study, we used either schizophrenia and personality disorder, which are risk factors for coercive measures (Beames and Onwumere, 2022) or other disorders (Baggio *et al.*, 2023) including dementia and cognitive disorders, mood disorders, neurotic and psychosomatic disorders, substance use disorders, intellectual disabilities); HoNOS at admission, which provided an indication of the severity of mental health problems at admission; and the first item on the HoNOS at admission, which assesses symptoms that signal hyperactive, aggressive, disruptive or agitated behaviour.

### Statistical analyses

We did not calculate sample size a priori. We calculated a sensitivity power analysis to determine the minimum effect size the study could detect, based on the smallest exposed group. Positing *n* = 3,589 in the restrained group, *n* = 204,200 in the unrestrained group, alpha = .05, power = .80 and a two-tailed independent *t*-test with a mean score of 11.2 in the control group, effect size was *d* = .05, so our study was powered to detect small effect sizes.

First, we calculated preliminary statistics for the whole sample. We then ran separate calculations for the primary exposure. To describe the variables, we used percentages and means. We then compared groups using absolute values ofstandardized mean/proportion difference (SMD).

Second, we used inverse probability (IP) of treatment weighting to emulate the trial, so we could balance the groups and create a pseudo-population in which the exposure was not associated with confounders (Hernán and Robins, 2016). We used stabilized weights. Briefly, we fitted a logistic regression with the exposure as the dependent variable and all confounders as covariates. The fitted values from this regression represented the denominator of the IP weights. Then we fitted a saturated model, with only the exposure as the dependent variable and no covariates. These fitted values represented the numerator of the IP weights. Because values were missing from discharge HoNOS, we used stabilized weights to account for attrition. The denominator was calculated using the fitted values of the logistic regression of all confounders on the binary variable ‘censored or not’. The numerator was determined by the fitted values returned by the logistic regression of the exposure on the binary variable ‘being censored or not’. To obtain the final IP weights, we multiplied these two weights. We derived IP weights separately for each exposure (primary and secondary exposures).

Third, we explored the effect of different coercive measures on mental health status. We used linear regression models; we predicted total HoNOS score at discharge with each exposure and used IP weight to control for confounding with robust standard errors. We used robust standard errors because anonymized data prevented us from identifying for patients with multiple hospital stays. We used Stata 18 for all analyses.

## Results

### Descriptive statistics

We included 207,789 psychiatric hospitalizations (records) in the study (104,224 participants). After excluding records with baseline missing values (*n* = 3,584, 1.7%), our final sample included 204,205 records (102,886 participants). At hospital discharge, HoNOS were completed for 178,369 records (retention rate = 87.4%). Table 2 shows the prevalence of coercive measures: 9.2% of the records had at least one coercive measure during hospitalization, most commonly seclusion (7.4%), forced medication (3.3%) or mechanical restraint (1.7%).

**Table 2.** Prevalence of coercive measures

Exposure	Prevalence (*n*)	95% CI
Coercive measures^a^	9.2 (18,800)	9.1; 9.3
Seclusion	7.4 (15,046)	7.3; 7.5
Restraint	1.7 (3,543)	1.7; 1.8
Forced medication	3.3 (6,727)	3.2; 3.4

CI: confidence interval.

a Include having at least one coercive measure during hospitalization (seclusion, restraint or forced medication).

Descriptive statistics and group comparisons for the primary exposure are shown in Table 3. The average age of participants was 46.3 years old; 52.8% were women. Absolute values of SMD between groups were large (≥0.50) or medium (≥0.30) for several variables. The highest was a mental health variable: item 1 of the HoNOS (SMD = 1.13), which is symptoms that signal hyperactive, aggressive, disruptive or agitated behaviour. The next highest were compulsory admission (SMD = 1.11), HoNOS at admission (SMD = 0.60) and primary psychiatric disorder (SMD = 0.41).

**Table 3.** Descriptive characteristics of the sample and comparisons between groups (n = 204,205)

			Coercive measures		
Variables		Overall	No	Yes	SMD
Age (mean)		46.3	46.1	48.2	0.11
Gender (%)					
	Women	52.8	53.7	45.1	0.17
	Men	47.2	46.3	54.9	
Nationality (%)					
	Swiss	78.9	79.1	77.1	0.05
	Other	21.1	20.9	22.9	
Civil status (%)					
	Single, divorced, widower	71.7	71.6	72.4	0.21
	Married or registered partnership	23.2	23.7	18.4	
	Unknown	5.1	4.7	9.2	
Twelve-month previous hospitalizations in psychiatry (%)					
	No	65.7	66.6	56.9	0.20
	Yes	34.3	33.4	43.1	
Psychiatric ward (%)					
	Adult	96.2	87.2	77.1	0.26
	Geriatrics	13.8	12.8	22.9	
Duration of hospitalization (%)					
	Less than 3 weeks	51.5	52.0	46.2	0.12
	3 weeks or more	48.5	48.0	53.8	
Primary psychiatric disorder (%)					
	Other disorders	73.3	75.0	56.1	0.41
	Schizophrenia, bipolar disorder, personality disorders	26.7	25.0	43.9	
Compulsory admission (%)					
	No	77.7	82.1	34.1	1.11
	Yes	22.3	17.9	65.9	
HoNOS at admission (0–48) (mean)		19.0	18.6	22.9	0.60
Item 1 of the HoNOS at admission (0–4) (mean)		1.26	1.13	2.56	1.13
HoNOS at discharge (0–48) (*n* = 16,732) (mean)		11.3	11.0	13.7	0.37

SMD: absolute values of standardized mean difference.

### Main results

Table 4 shows the results for the primary and secondary exposures after trial emulation, balancing the groups’ differences highlighted above. Each exposure had significant effects on total HoNOS score at discharge. Participants with any type of coercive measure during hospitalization (primary exposure) had a 1.91-point higher score than those with no coercive measure (*p* < .001). Results were similar for seclusion (1.60-point higher score, *p* < .001) and forced medication (1.97-point higher score, *p* < .001). The effect was stronger for restraint (2.83-point higher score, *p* < .001, non-overlapping confidence intervals with other coercive measures). Overall, effects were small.

**Table 4.** Estimation of the effect of coercive measures on the HoNOS score

		Crude			Adjusted with IPW		
Exposures		*b*	95% CI	*p*	*b*	95% CI	*p*
Primary exposure							
	Any coercive measure^a^	2.65	2.53; 2.77	<.001	1.91	1.73; 2.09	<.001
Secondary exposures							
	Seclusion	2.19	2.05; 2.32	<.001	1.60	1.40; 1.79	<.001
	Restraint	4.62	4.35; 4.88	<.001	2.83	2.38; 3.28	<.001
	Forced medication	2.04	1.84; 2.24	<.001	1.97	1.65; 2.30	<.001

HoNOS: Health of the Nations Outcome Scale, IPW: inverse probability weighting, *b*: estimate, CI: confidence interval.

a Seclusion, restraint or forced medication.

## Discussion

This study emulated a trial to investigate the effects of coercive measures on the mental health status of psychiatric inpatients at discharge. The primary exposure was a composite of receiving any type of coercive measures (seclusion, restraint or forced medication) during hospitalization. Secondary exposures separately examined the effects of seclusion, restraint and forced medication.

### Summary of main findings

In our trial emulation, use of coercive measures was significantly associated with worse mental health status at discharge. Mental health problems increased by 1.91 points for the primary exposure of any type of coercive measure, 1.60 for seclusion, 2.83 for restraint and 1.97 for forced medication over those of participants who did not receive the same coercive measure. Our findings align with findings of earlier studies that suggested seclusion and restraint degraded patient health (Baggio *et al.*, 2023; Chieze *et al.*, 2019), but ours is the first large-scale study to show the deleterious effect of coercion on mental health using a robust method. This is also in line with previous study findings showing harmful mental health consequences of the use of coercive measures, such as post-traumatic symptoms (Steinert *et al.*, 2013; Whitecross *et al.*, 2013; Wullschleger *et al.*, 2021).

Our results suggest that the negative effects of restraint were greater than of seclusion and forced medication, in line with previous research findings. A systematic review had reported that patients found restraint less acceptable than seclusion and forced medication, probably because restraint was invasive, but these studies often focused on a single coercive measure (Chieze *et al.*, 2019). Previous studies found negative emotions, traumatization, the patient’s negative evaluation of the benefits of the intervention and perceived coercion were associated with worse outcomes (Chieze *et al.*, 2019; Georgieva *et al.*, 2012; Guzmán-Parra *et al.*, 2019; Steinert *et al.*, 2013). However, restraints are rarely used in the Swiss healthcare system and are likely to be used for specific patients, such as those with severe psychotic symptoms, aggressive behaviour or uncooperative behaviour. Nevertheless, it is important to show that restraint can also have a detrimental effect on their mental health problems.

We also found that forced medication is associated with worse mental health, which has been understudied (McLaughlin *et al.*, 2016; Muir-Cochrane *et al.*, 2020). Forced medication has already been associated with stronger disapproval of treatment at 3-month follow-up than other coercive measures (McLaughlin *et al.*, 2016). Forced medication may raise concerns in a different subgroup of patients than those subject to seclusion or mechanical restraint (Meroni *et al.*, 2023). We need to better understand how patients experience coercive measures at a range time points after they were implemented.

However, the results should be interpreted in the light of two important drawbacks. First, although the effects of exposure were significant, they were small. Total HoNOS score can range from 0 to 48, and the differences we identified were only 2–3 points. Another study that focuses on seclusion in a Swiss university hospital also found small effects (Baggio *et al.*, 2023). There is no consensus on the clinical relevance of HoNOS changes. Most studies investigating this question have focused on intra-individual changes, usually over the course of treatment, rather than between-individual or between-group comparisons as used here. Also, the number and duration of coercive measures were not examined, which may have minimized the magnitude of potential effects. But just because effects are small, this does not mean they are negligible: coercive measures, with and without therapeutic purpose, are neither therapeutic nor neutral interventions. The finding that coercive measures can harm patients should challenge the belief of some healthcare professionals who believe that coercive measures, especially forced medication, can yield therapeutic benefits (Chieze *et al.*, 2019; Doedens *et al.*, 2019). Second, even if we used a causal analytical framework to test whether coercive measures worsen mental health, we cannot exclude the possibility that worsening mental health led to a greater likelihood of using coercive measures. In addition, coercive measures may be an indicator of treatment failure (e.g., increase in symptom severity, lack of treatment adherence, external interference). These time-varying factors were not assessed in the study, so caution is needed when interpreting our results.

### Clinical implications

The potentially harmful consequences of coercive measures highlighted by our findings reinforce the need for a rigorous ethical and clinical decision-making process to ensure that such measures are used only when all other alternatives have been tried. Their use should follow a principle of proportionality in terms of the type of measures used and their duration. Their use must involve the provision of intensive psychiatric care, considering the needs of the patient. In this context, post-coercion review must be considered mandatory, as it has been shown to have positive effects in mitigating some of the consequences of coercive measures on perceived coercion and post-traumatic stress disorder symptoms (Wullschleger *et al.*, 2021).

Clinicians might underestimate the potential harmful effects of coercive measures when they make treatment decisions. Alternatives to coercive measures are often not well integrated into clinical practice, and patients and professionals may see these alternatives differently (Heumann *et al.*, 2021). While patients may prefer alternatives, healthcare staff members may consider them insufficient or impossible to implement with available resources. When feasible alternatives are not readily available and the harmful consequences of coercion are underestimated, coercive measures may become routine instead of being a true ‘last resort’. The fact that mechanical restraint seems to be the most harmful coercive measure should lead to a stronger limitation of its use.

Staff should be educated about the negative consequences of coercive measures, should be shown the scientific evidence and should also be shown patients’ first-hand reports about the effects of coercion. Researchers and clinicians should develop, test and implement alternatives to coercive measures, such as intensive one-to-one interventions, use of sensory rooms or joint crisis plans. Such strategies and alternatives have been the subject of recent recommendations (Steinert and Hirsch, 2020). Organizational models of inpatient care, such as the Six Core Strategies, Safewards or the Weddinger Model, which include multilevel interventions focused on patient-centred care, transparency and participation, should be promoted to reduce the use of coercion (Johnson *et al.*, 2022).

### Limitations

This study had three main limitations. First, we cannot exclude the existence of a reverse causal relationship (i.e., deteriorating mental health leads to an increased likelihood of coercive measures). Even if our study relied on a robust analytic approach and included the key factors in predicting use of coercive measures, it may suffer from unmeasured confounding. We only included baseline confounders; future studies would benefit from including time-varying confounders, such as therapeutic adherence, evolution of symptoms over time, and other relevant patient and external factors, or more specific baseline variables, such as substance use. Second, we could not reliably assess the number and duration of coercive measures, so we had to limit our analysis to simple exposure. Researchers who conduct prospective studies should agree on standards for measuring coercive measures so we can accurately capture the effect of their intensity on patients’ mental health. We suggest guidelines for careful documentation of coercive measures (e.g., reasons, circumstances) whenever they are used (Luciano *et al.*, 2018). Third, we based our analysis on data gathered between 2019 and 2021, much of it during the COVID-19 pandemic. Though the pandemic was associated with an increase in use of coercive measures (Wullschleger *et al.*, 2023), we think it unlikely this relative increase significantly changed the clinical consequences of coercive measures. Finally, the study did not capture the long-term detrimental effects of coercive measures on mental health. There is a need for further prospective studies of the long-term effects of coercion, including the use of instruments that might better capture long-term patient outcomes.

### Conclusions

Our study presents robust empirical evidence highlighting the detrimental impact of coercive measures, such as seclusion, restraint and forced medication, on the mental health of psychiatric inpatients. It underscores the importance of avoiding these measures in psychiatric hospitals due to their small but not negligible effect on patients’ mental health. This research emphasizes the urgent need for implementing alternatives in clinical practice and providing staff education to prevent the overuse of coercive measures.

## Data Availability

We extracted our data from a national registry, which is only accessible only by request for specific scientific projects, subject to approval by the Swiss National Association for Quality Development in Hospitals and Clinics (ANQ).
